# Persian Mentalized Affectivity Scale (MAS): Reliability, Validity, and Cultural Considerations

**DOI:** 10.1002/brb3.70880

**Published:** 2025-09-21

**Authors:** Fateme Jafarpoor, Elmira Shayegh, Parvin Haghjoo, Saeed Ghanbari

**Affiliations:** ^1^ Faculty of Education and Psychology Shahid Beheshti University Tehran Iran

**Keywords:** Confirmatory Factor Analyses (CFA), Exploratory Factor Analyses (EFA), mentalization, mentalized affectivity, Mentalized Affectivity Scale (MAS), Persian version

## Abstract

**Introduction:**

Mentalization and emotion regulation are increasingly recognized as key factors in the development and persistence of psychological disorders. Mentalized affectivity, an integrative construct encompassing both, reflects an individual's capacity to reflect upon and regulate emotions. Despite the growing interest in this construct, cross‐cultural validation of its measurement remains limited. This study aimed to examine the psychometric properties of the Persian version of the Mentalized Affectivity Scale (MAS) in an Iranian adult sample.

**Methods:**

A total of 923 participants were recruited through convenience sampling and divided into two independent samples (*N*_1_ = 473, *N*_2_ = 450). In addition to the MAS, measures assessing emotion regulation and reflective functioning were administered in the second sample to evaluate concurrent validity. Exploratory factor analysis (EFA) and confirmatory factor analysis (CFA) were conducted to determine the factorial structure of the MAS. Internal consistency and correlation analyses were performed to assess reliability and construct validity.

**Results:**

EFA yielded a four‐factor model, encompassing Identifying Emotions, Processing Emotions, Expressing Emotions, and Recognizing Emotions. This structure diverges from the original three‐factor model, potentially reflecting cultural distinctions in emotional processing. The strongest correlation coefficients were observed between Processing and Recognizing Emotions, which were previously merged as a single factor in the original scale. Internal consistency was excellent, with Cronbach's alpha values ranging from 0.847 to 0.895 and McDonald's omega ranging from 0.831 to 0.904, confirming the reliability of the Persian MAS.

**Conclusion:**

The findings provide preliminary validation for the Persian version of the MAS, supporting its factorial validity, reliability, and applicability for assessing mentalized affectivity in nonclinical populations. Given the scarcity of culturally adapted measures of mentalization in Iran, the MAS represents a valuable tool for both research and clinical settings.

## Graphical abstract

The Persian Mentalized Affectivity Scale (MAS) was validated in an Iranian adult sample (*N* = 923), confirming a four‐factor structure: Identifying, Processing, Expressing, and Recognizing Emotions. Strong correlations were observed between Processing and Recognizing (*r* = 0.518), and Identifying and Recognizing (*r* = 0.430). Reliability was excellent (*α* = 0.847–0.895; *ω* = 0.831‐0.904), and model fit indices (CFI = 0.91, RMSEA = 0.075, SRMR = 0.090) supported validity. Findings suggest MAS as a robust tool for assessing mentalized affectivity in Iranian contexts.

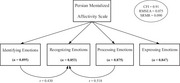

## 1 Introduction

At times, individuals may not be fully aware of their emotions; this phenomenon has been widely discussed in the literature on emotion and affect (Jurist 2018; Ivonin et al. 2013). Emotions are a core part of subjective experience and develop from nonverbal communication in infancy to more complex expressions in adulthood, supported by strategies that enable effective functioning in the social realm (Jurist 2005; Greenberg et al. 2017). These strategies contribute to emotion regulation, sometimes referred to as affect regulation, aimed at processing emotional states, modulating their expression and evaluation (Schwarzer et al. 2021). In contemporary psychology, emotion regulation is commonly defined as the cognitive and behavioral management of emotional responses. In contrast, affect regulation, grounded in psychoanalytic and developmental theories, refers to broader modulation of enduring affective states, including physiological and preconscious processes. From an attachment and psychoanalytic perspective, advanced regulation extends beyond modifying affects to regulating the self (Gross 2015). However, existing measures of emotion regulation have primarily emphasized dysfunctions rather than their role in self‐regulation (Greenberg et al. 2017; Fonagy et al. 2018). Recent research further distinguishes explicit from implicit (automatic) emotion regulation. Meta‐analytic and clinical neurophysiology evidence indicates that explicit strategies recruit fronto‐parietal control networks. In contrast, implicit regulation reduces negative affect and modulates amygdala/insula activity with minimal engagement of higher‐order control (Pozzi et al. 2021; Yuan et al. 2023).

Mentalization, a higher‐order developmental capacity, is defined as “the imaginative capacity to perceive and interpret one's own and other's behaviors in terms of intentionally motivated mental states—such as feelings, wishes, desires, thoughts, and beliefs” (Fonagy et al. 2018)—and it serves as the reflective framework within which affect‐regulation processes are implemented and coordinated. Mentalization, which is central to human social functioning (Fonagy 2011) and have evolved from the earliest relationship with a primary attachment figure (Fonagy and Allison 2013), makes us capable of reflecting on our and others' mental states, interpreting them, and understanding each other's intentions (Devaine et al. 2014; Frith and Frith 2003). Given the importance of mentalization as a “health promotion resource” (Schwarzer et al. 2021) and its evident traces in stress regulation (Nolte et al. 2011), an increasing number of studies in developmental psychology and clinical practice suggest that impairments in different dimensions of mentalization can be found in a variety of psychopathologies, including borderline personality disorder (Fonagy and Bateman 2008), schizophrenia (Das et al. 2012), eating disorders (Kelton‐Locke 2016), antisocial personality disorder (Newbury‐Helps et al. 2017), functional somatic disorders (Luyten et al. 2012), and depressive disorders (Fischer‐Kern and Tmej 2019).

From a developmental perspective, the capacity to regulate affects is achieved through a relationship with a sensitive and responsive caregiver (Cassidy 1994; Thompson 1994). A secure attachment opens the door to affect regulation, which contributes to the transformation of self‐regulation from the state of co‐regulation between infant and caregiver (Evans and Porter 2009). With the development of a capacity for regulating affects, a growing sense of self arises and paves the way for a more sophisticated regulation (Fonagy et al. 2018; Jurist 2005). The process of regulating affects entails recognizing intentions and mental states in others and also in oneself as an agent, engendering mentalization (Sharp et al. 2011). Fonagy et al. (2018) proposed that affect regulation provides the developmental foundation for the emergence of mentalization. However, subsequent infant research demonstrates that proto‐mentalizing capacities can already be observed in the second year of life—well before children pass standard false‐belief tasks—indicating a reciprocal and dynamic developmental interplay between affect regulation and mentalization (Király et al. 2013). As mentalization emerges, affect regulation transforms into a more sophisticated form of affect and self‐regulation that is called “mentalized affectivity,” where not only affects are modulated, but it also serves as a self‐regulating capacity (Jurist 2005).

Mentalized affectivity, which was coined by Jurist (2005), characterizes an elaborate capacity to reflect and re‐evaluate one's emotions. It involves reappraising affects rather than just regulating them. It clarifies how our representational world and past experiences, whether arising from our fantasy or external reality, influence the affective quality of our present and future experiences. In other words, “there are often affects behind affects” (Jurist 2005, 2010). Mentalized affectivity leads us to attain a more complicated appreciation of an affective experience. As a result, it gives rise to positive affects and containment of and coping with negative affects (Jurist 2018). According to the Mentalized Affectivity model, an affective regulatory process has three elements: “Identifying emotions,” “Processing emotions,” and “Expressing emotions.” Each of these elements can be simple or complex. Presumably, for adaptive affect regulation, one needs to have awareness of that emotional experience. This awareness entails the ability to distinguish and label that emotion, but it does not end here. In its complicated form, identifying emotions enables the person to gain insight into the deeper meaning of subjective experience. Following Identifying emotions is “Processing emotions,” which is modulating and regulating them. Encountering an unmentalized emotion, to regulate it, one has to make modulations in different aspects of that emotion, such as its intensity or duration. The last process that follows processing is “Expressing emotions,” which involves inward and outward communication of affects (Jurist 2005).

Mentalized Affectivity has been operationalized via the Mentalized Affectivity Scale (MAS; Greenberg et al. 2017), which is a 60‐item self‐report measure evaluating components of Mentalized Affectivity (identifying, processing, and expressing emotions). These components have been demonstrated to be linked to different psychological constructs, such as well‐being, trauma, mood disorders, personality disorders, and neurobiological disorders (Greenberg et al. 2017).

Recently, a considerable body of cross‐cultural studies on emotion regulation and mentalization has been conducted, indicating that there are cultural differences in emotion regulation and mentalization processes (e.g., Aival‐Naveh et al. 2019). Hence, considering a cultural perspective when assessing these constructs seems mandatory. Regarding emotion regulation, Asian societies tend to suppress their emotions (Butler et al. 2007; Kitayama et al. 2006), while in European societies, the expression of emotions is highly valued and desirable (Mauss and Gross 2004; Murata et al. 2013). Ma et al. (2018) found that people in Asia process emotions differently from people in Western countries. Aival‐Naveh et al. (2019) employed a cross‐cultural lens to review the concept of mentalization. They also conducted a comprehensive and systematic review of five psychological constructs that overlap with mentalizing. The findings of this review raised profound implications for the study of mentalization: we cannot simply generalize findings from mentalization studies without considering substantial cultural variations. For instance, in collectivistic cultures, there is a marked tendency toward understanding others' mental states rather than oneself; meanwhile, in individualistic cultures, focusing on one's mental state is far preferable to attending to other people's internal worlds (Aival‐Naveh et al. 2019). Jurist and Sosa (2019), in their commentary on this review, propounded that in the ongoing process of validating the MAS in other languages and according to the feedback of translators from different cultures, it was obvious that there is a need to take these cultural aspects into further consideration.

In Iran, scholarly attention to mentalization and its related constructs has been steadily increasing in recent years (e.g., Ghanbari et al. 2022; Vahidi et al. 2021). Building on this emerging body of research, the present study focuses on mentalized affectivity, a construct that integrates reflective functioning with emotion regulation. Specifically, we aim to examine the psychometric properties of the Persian version of the MAS in a representative sample of Iranian adults. Guided by the original validation of the MAS (Greenberg et al. 2017) and its theoretical foundations, we formulated three hypotheses. First, we anticipated that the Persian version of the MAS would replicate the original three‐factor structure—Identifying, Processing, and Expressing emotions—while also allowing for the possibility that cultural particularities in emotional experience might give rise to alternative structures. Second, we expected the MAS subscales to demonstrate robust internal consistency, with Cronbach's alpha and McDonald's omega coefficients exceeding 0.80. Finally, we hypothesized that the MAS subscales would show significant positive correlations with established measures of reflective functioning and emotion regulation, thereby providing evidence for convergent validity.

## 2 Methods

### 2.1 Participants

Two independent samples comprised 450 participants (317 (71.1%) female; mean age = 27.47; SD = 8.68; range 14–65 years) and 473 participants (344 (72.7%) female; mean age = 25.19; SD = 6.59; range 15–55 years) were formed for exploratory and confirmatory analyses. Educational attainment in the combined sample was: 18 (2%) without a diploma, 227 (24.6%) with a diploma, 128 (13.9%) with a college degree, 233 (25.2%) with a BSc, 257 (27.8%) with an MSc, and 60 (6.5 %) with a PhD.

### 2.2 Procedure

The Persian translation of the MAS followed an eight‐step cross‐cultural adaptation protocol (Cruchinho et al. 2024): two independent forward translations by bilingual psychologists, synthesis into a reconciled version, blind back‐translation by an English native speaker fluent in Persian, expert‐panel harmonization (translators, clinical psychologists, and psychometricians), pilot pretesting with cognitive feedback from the target population, finalization, and psychometric validation. Steps 7 and 8 comprised the field testing and statistical validation reported here. Field data collection was conducted online between December 2023 and February 2024 using the Porsline as a favorite online survey platform in Iran. Recruitment employed a convenience/snowball procedure: initial “seed” invitations were distributed by the research team via social media and professional/educational networks, and recipients were invited to complete the survey and to share the survey link with their contacts. Therefore, the sample is not population‐representative; it shows an overrepresentation of women and individuals with higher education relative to the general Iranian population.

On the survey's first page, participants received an information sheet describing study aims, estimated completion time, data handling, and confidentiality; only those who provided informed consent could proceed. No monetary compensation was offered; participation was voluntary and anonymous. Participants completed sociodemographic items first, followed by the Persian MAS, the emotion regulation questionnaire (ERQ; Gross and John 2003), and the reflective functioning questionnaire (RFQ; Fonagy et al. 2016) for concurrent validity assessment. The platform recorded completion time (mean ≈ 19 min). To reduce duplicate or low‐quality entries, we applied standard data‐cleaning procedures: duplicate IPs and identical response patterns were examined and, where appropriate, removed; attention‐check items were embedded, and failed cases were excluded from final analyses. All procedures in the current study were approved by the Research Ethics Committee at Shahid Beheshti University and were conducted in accordance with the principles outlined in the Declaration of Helsinki.

### 2.3 Measures

#### 2.3.1 Sociodemographic Characteristics

All participants were asked to provide sociodemographic information, including gender, age, education level, the field of study, marital status, and the number of children.

#### 2.3.2 Mentalized Affectivity

The MAS is a 60‐item self‐report measure that was introduced by Greenberg et al. (2017) to assess mentalized affectivity. This measure evaluates Mentalized Affectivity on a seven‐point Likert scale, from 1 (*strongly disagree*) to 7 (*strongly agree*). In this scale, 24, 23, and 13 items represent Identifying emotions, Processing emotions, and Expressing emotions, respectively, as three factors of Mentalized Affectivity. Calculated Cronbach's *α* values of 0.93, 0.90, and 0.88 for identifying, processing, and expressing emotions, respectively, show that these subscales of the MAS have satisfactory internal consistency (Greenberg et al. 2017). In the Italian validation, Rinaldi et al. (2021) identified a five‐factor structure that includes identifying emotions, expressing emotions, curiosity about emotions, processing emotions, and autobiographical memory. This structure differs from the original three‐factor model (Greenberg et al. 2017) by adding two distinct dimensions; curiosity and autobiographical memory.

#### 2.3.3 Emotion Regulation

The ERQ is a self‐report 10‐item measure that evaluates emotional regulation (Gross and John 2003). It includes a seven‐point Likert scale from 1 (*strongly disagree*) to 7 (*strongly agree*), with minimum and maximum scores ranging from 10 to 70. ERQ is comprised of two dimensions: emotion regulation strategies of cognitive reappraisal (six items) and expressive suppression (four items). It has been shown that both reappraisal and suppression subscales of the ERQ have good internal consistency, with Cronbach's *α* values of 0.79 and 0.73, respectively. Hasani (2016) developed the Persian version of ERQ (ERQ‐P) and found satisfactory internal consistency for both reappraisal and suppression subscales with Cronbach's *α* values of 0.87 and 0.85, respectively. In the present study, the ERQ‐P measure is utilized, and Cronbach's *α* for the cognitive reappraisal subscale is 0.85, and for the expressive suppression subscale is 0.766.

#### 2.3.4 Reflective Functioning

The RFQ is a self‐report eight‐item measure that evaluates reflective functioning as a single, global dimension of certainty/uncertainty about mental states (Fonagy et al. 2016). This questionnaire uses a seven‐point Likert scale, from 1 (*strongly disagree*) to 7 (*strongly agree*). Although the original model proposed two subscales—certainty (RFQc) and uncertainty (RFQu)—recent researches and psychometric considerations support the use of an aggregated total score when the primary focus is on overall reflective functioning rather than fine‐grained dimensional distinctions. The Persian translation of the RFQ demonstrated satisfactory internal consistency for the total score, with Cronbach's *α* = 0.77 (Seyed Mousavi et al. 2021). In the present study, we adopted this unidimensional approach and calculated the total RFQ score, which showed good internal consistency (Cronbach's *α* = 0.761).

### 2.4 Statistical Analysis

Data were screened for normality and outliers. Univariate distributions of all factor scores were examined through skewness and kurtosis, with values ranging from −0.818 to −0.200 for skewness and −0.194 to 1.072 for kurtosis, indicating no substantial deviations from univariate normality. Multivariate outliers were assessed using Mahalanobis distances (*χ* ^2^, *p* < 0.001 criterion). No cases exceeded the critical value; therefore, no participants were excluded. There was no missing data in the final datasets because the online survey required completion of all items before submission. Therefore, no imputation or other missing‐data procedures were necessary. Given these results and the relatively large sample size, the maximum likelihood (ML) estimation method was selected for assessing the parameters of the confirmatory factor analyses (CFA) models.

Exploratory factor analysis (EFA) was conducted on Sample 1 (*N*_1_ = 473) using Principal Axis Factoring with varimax rotation in IBM SPSS Statistics 25. The suitability of the data for EFA was assessed by calculating the Kaiser–Meyer–Olkin Test for sample adequacy. Moreover, by assessing the intercorrelation of the items, Bartlett's test of Sphericity evaluated the data's potential for factor extraction (*p* < 0.05 level) (Tabachnick and Fidell 2019). Considering the sample size (*N* > 350), a factor loading of 0.3 was selected as the cut‐off value (Hair et al. 2019). In addition, items showing cross‐loadings with a primary‐to‐secondary loading ratio of less than 1.5 and those that loaded substantially on method factors were flagged for exclusion. No items with low loadings but high theoretical importance were observed in the current data, and therefore, no such items were retained. Parallel analysis was performed as the best criterion for choosing the number of factors. Internal consistency of each MAS subscale was assessed using Cronbach's *α* and McDonald's *ω*. Also, we computed average variance extracted (AVE) and composite reliability (Jöreskog's rho) for each factor.

CFA were performed on Sample 2 (*N*_2_ = 450) to validate the EFA‐derived model and to compare it with alternative specifications (original English three‐factor model and Italian five‐factor model). Model fit was evaluated with the chi‐square statistic (*χ* ^2^) and its degrees of freedom, the comparative fit index (CFI), the Tucker–Lewis Index (TLI), the root mean square error of approximation (RMSEA) with its 90% confidence interval, the standardized root mean square residual (SRMR), the Akaike information criterion (AIC), and the Bayesian information criterion (BIC). Furthermore, the convergent validity of the questionnaire was examined by calculating the Pearson correlation. Error covariances were not freely estimated in any of the CFA models reported here. Potential correlated residuals were inspected using modification indices; however, no error covariances met both (a) substantive/theoretical justification, and (b) sufficient statistical indication to warrant inclusion, and therefore none were added to the models. To obtain error‐adjusted associations between MAS factors and external constructs, we estimated an SEM in which the four‐factor MAS measurement model (52 indicators) was retained as validated and external measures were modeled as single‐indicator latent variables. We reported standardized latent correlations (*φ*) with 95% CIs. As a sensitivity analysis, ERQ was also modeled with its two latent factors (expressive suppression and cognitive reappraisal); the pattern of results was unchanged.

## 3 Results

### 3.1 Exploratory Factor Analysis

**FIGURE 1:**
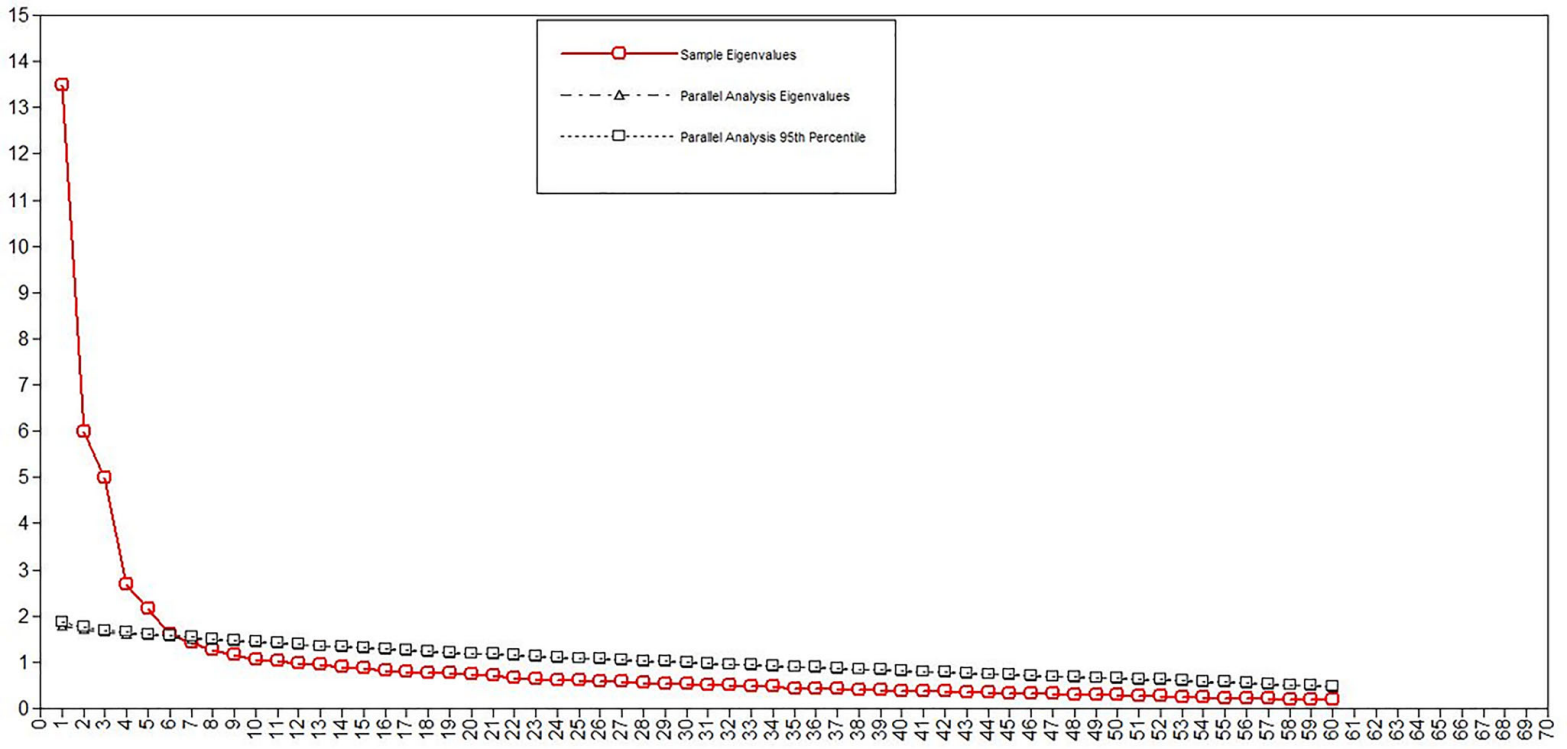
Parallel analysis of the MAS items (*N*_1_ = 473).

Using a sample of 473 participants, the KMO index was 0.916 and Bartlett's test of sphericity was statistically significant (*p* < 0.01, *𝑥* ^2^ = 14,889.050), confirming the suitability of the data for factor analysis. Initial EFA, guided by Kaiser–Guttman's criterion and Cattell's scree test, suggested 11 factors. However, a subsequent parallel analysis (Figure 1) indicated a more parsimonious five‐factor model.

More attention to the items dedicated to these five factors revealed one “method factor” among them, which included reverse‐keyed (RK) questions. Although such items are often included to mitigate response bias (van Sonderen et al. 2013), prior work has shown that they may reduce internal consistency and validity due to inattentive responding or cognitive fatigue (Woods and Rodebaugh 2005; Woods 2006; Merritt 2011). To refine the structure, EFA was rerun with four factors after excluding the RK method factor. A stepwise elimination process was then applied: items with loadings < |0.30| (Items 40 and 53) and items with problematic cross‐loadings (Items 12, 8, 19, 32, 13, and 55) were removed. The final model comprised 52 items across four substantive factors (Table 1). These factors accounted for 47.318% of the total variance, with eigenvalues ranging from 2.318 to 12.380. Although the AVE values fell below the conventional Fornell–Larcker threshold (0.50), the scale demonstrated strong composite reliability and theoretically coherent factor composition. Thus, the four‐factor Persian MAS shows acceptable construct validity for this cultural context.

**TABLE 1:** Component loading for the 52‐item MAS.

	Factors			
Item	Identifying	Processing	Expressing	Recognizing
54‐ It's important to understand the major life events that have had an impact on my behavior.	**0.731**			
42‐ I am curious about identifying my emotions.	**0.697**			
51‐ It is helpful to think about how my emotions stem from family dynamics.	**0.683**			
46‐ I try to put effort into identifying my emotions.	**0.644**			
34‐ It is important for me to acknowledge my own true feelings.	**0.638**			
16‐ Knowing about my childhood experiences helps to put my present emotions within a larger context.	**0.635**			
37‐ I often look back at my life history to help inform my current emotional state and situation.	**0.611**			
60‐ I am interested in learning about why I feel certain emotions more frequently than others.	**0.596**			
50‐ I can see how prior relationships influence the relationships that I have now.	**0.592**			
28‐ It helps me to know the reasons behind why I feel the way that I do.	**0.586**			
47‐ I can pinpoint childhood experiences that influence the way that I often think and feel.	**0.577**			
5‐ I can see how prior relationships influence my current emotions.	**0.554**			
33‐ I try to understand the complexity of my emotions.	**0.550**			
49‐Thinking about other people's emotional experiences helps me to think about my own.	**0.538**			
1‐ I often think about how the emotions that I feel stem from earlier life experiences (e.g. family dynamics during childhood).	**0.511**			
18‐ I often think about my past experiences to help me understand emotions that I feel in the present.	**0.498**	0.313		
52‐ I am open to other people's view of me because it helps me to better understand myself.	**0.462**			
29‐ I am aware of recurrent patterns to my emotions.	**0.417**			0.343
38‐ I am open to what others say about me to help me know what I am feeling.	**0.409**			
3‐ I am good at understanding other people's complex emotions.	**0.342**			
23‐ I am good at controlling my emotions.		**0.778**		
10‐ When I am filled with a negative emotion, I know how to handle it.		**0.767**		
14‐ I am able to adjust my emotions to be more precise.		**0.745**		
26‐ I am good at controlling emotions that I do not want to feel.		**0.690**		
43‐ If a feeling makes me feel uncomfortable, I can easily get rid of it.		**0.644**		
15‐ It is hard for me to manage my emotions.		**0.594**		
7‐ I am able to wait to act on my emotions.		**0.582**		
6‐ I can still think rationally even if my emotions are complex.		**0.565**		
25‐ When I am filled with a positive emotion, I know how to keep the feeling going.		**0.534**		
4‐ I use tools I have learned to help when I am in difficult emotional situations.	0.301	**0.499**		
22‐ I am good at increasing emotions that I want to feel more.		**0.477**		
20‐ I often keep my emotions inside.			**0.754**	
31‐ If I feel something, I prefer not to discuss it with others.			**0.691**	
44‐ I often know what I feel but choose not to reveal it outwardly.			**0.691**	
48‐ If I feel something, I will convey it to others.			**0.688**	
36‐ If I feel something, I rather not convey it to others.			**0.674**	
27‐ I am quick to act on my emotions.			**0.666**	
2‐ I can express my emotions clearly to others.			**0.606**	
30‐ People tell me I am good at expressing my emotions.			**0.589**	
9‐ It is hard for me to talk about my complex emotions.			**0.564**	
56‐ I am more comfortable “talking around” emotions I am feeling, rather than talking about them directly.			**0.444**	
45‐ If I feel something, it often comes pouring out of me.		−0.369	**0.423**	
24‐ When I express my emotions to others, it is usually jumbled.			**0.393**	
39‐ People get confused when I try to express my emotions.			**0.381**	
59‐ I can tell if I am feeling a combination of emotions at the same time.				**0.781**
41‐ I am good at distinguishing between different emotions that I feel.				**0.777**
58‐ I am able to understand my emotions within the context of my surroundings.				**0.657**
57‐ I can quickly identify my emotions without having to think too much about them.				**0.650**
21‐ I can easily label “basic emotions” (fear, anger, sadness, joy, and surprise) that I feel.				**0.642**
35‐ I often figure out where my emotions stem from.	0.350	0.319		**0.492**
17‐ It is easy for me to notice when I am feeling different emotions at the same time.				**0.470**
11‐ I often know the reasons why I feel the emotions I do.		0.317		**0.433**
				
Eigenvalues	12.380	5.415	4.492	2.318
% of variance	14.231	11.081	9.955	8.007
Cronbach's *α* (Total = 0.886)	0.895	0.875	0.847	0.853
McDonald's *ω* (Total = 0.910)	0.904	0.878	0.872	0.831
AVE	0.327	0.401	0.342	0.420
Jöreskog's rho (CR)	0.904	0.878	0.874	0.831
Identifying	1			
Processing	0.228^**^	1		
Expressing	0.056	−0.104^*^	1	
Recognizing	0.430^**^	0.518^**^	0.116^*^	1

*p < 0.05.

**p < 0.01.

### 3.2 Confirmatory Factor Analysis

CFA was carried out with LISREL 10.3, using the maximum likelihood estimation method on the second sample (*N*_2_ = 450) to validate the factorial structure derived from EFA and to compare it with alternative factor structures. Three models were tested: The Persian four‐factor model obtained from the current EFA after item reduction (52 items), the original English three‐factor model, and the adapted Italian five‐factor model. Table 2 presents the fit indices and information criteria (AIC, BIC). Adequate model fit was determined using established cutoffs (RMSEA < 0.08; CFI > 0.90; TLI > 0.90; SRMR < 0.10). Lower AIC and BIC values were considered indicative of more parsimonious models.

**TABLE 2:** Obtained goodness‐of‐fit indices and information criteria (AIC, BIC) for three MAS models tested with the Persian dataset.

Model		Model Fit Indices									Model fit comparisons				
	structure	*χ* ^2^	*df*	CFI	TLI	RMSEA	CI 90%	SRMR	AIC	BIC	Δ*χ* ^2^	Δ*df*	ΔCFI	ΔTLI	ΔRMSEA
Persian	4	4439.63	1268	0.91	0.90	0.075	0.072–0.078	0.090	4659.63	5117.01					
Original English	3	8608.14	1707	0.87	0.86	0.095	0.092–0.098	0.110	8854.14	9365.57	4168.51	439	−0.04	−0.04	0.020
Italian	5	1852.57	517	0.92	0.91	0.076	0.073–0.079	0.088	3574.57	7151.31	−2586.06	−751	0.01	0.01	−0.001

The Persian four‐factor model demonstrated acceptable fit to the data (χ^2^ (1268) = 4439.63, *p* < 0.001; CFI = 0.91; TLI = 0.90; RMSEA = 0.075, 90% CI [0.072, 0.078]; SRMR = 0.090), outperforming both the original English three‐factor and the Italian five‐factor models. Importantly, this model also yielded the lowest BIC, suggesting superior efficiency in representing the latent structure within this cultural context. No residual covariances were freely estimated. Modification indices were inspected, but none provided sufficient statistical and theoretical justification to warrant inclusion. The final CFA specified four correlated first‐order factors with 52 indicators. A complete path diagram with standardized loadings is provided in Figure S1.

### 3.3 Reliability of the MAS and Correlations Among Factors

Cronbach's *α* values were: Identifying = 0.895, Processing = 0.875, Expressing = 0.847, Recognizing = 0.853. McDonald's *ω* values were: Identifying *ω* = 0.904, Processing *ω* = 0.878, Expressing *ω* = 0.872, and Recognizing *ω* = 0.831. These *ω* coefficients confirm acceptable‐to‐excellent scale reliability and provide a factor‐model consistent estimate of internal consistency; differences between *α* and *ω* likely reflect departures from tau‐equivalence among items (Dunn et al. 2014). Regarding correlations among the four factors, Identifying and Expressing were not associated, Processing and Expressing were negatively correlated, and all the other factors yielded significantly positive correlations.

### 3.4 Convergent Validity

Table 3 shows the bivariate correlations between reflective functioning (total score), emotion regulation, and the MAS subscales (*N*_2_ = 450). Table 4 further summarizes standardized latent correlations (*φ*) between MAS factors and external constructs. The latent estimates mirrored the direction and relative ordering of the raw‐score correlations, with modest increases in magnitude consistent with attenuation correction. Associations with ERQ Suppression remained negative for Expressing, whereas Identifying/Processing/Recognizing showed positive relations with Reflective Functioning and ERQ Reappraisal. Sensitivity analyses using the two‐factor ERQ specification yielded convergent conclusions.

**TABLE 3:** Bivariate correlations between reflective functioning (total score), emotion regulation, and the MAS subscales (*N*_2_ = 450).

Scale	MAS			
	Identifying	Processing	Expressing	Recognizing
RFQ‐ Total score	0.181^**^	0.392^**^	0.065	0.339^**^
ERQ‐ Expressive suppression	−0.091	0.103^*^	−0.705^**^	−0.099^*^
ERQ‐ Cognitive reappraisal	0.178^**^	0.423^**^	−0.132^**^	0.223^**^

*p < 0.05.

**p < 0.01.

**TABLE 4:** Standardized latent correlations (*φ*) between MAS factors and external constructs (*N*_2_ = 450).

Subscale	Identifying	Processing	Expressing	Recognizing
RFQ‐ Total score	0.22^**^	0.41^**^	0.05	0.36^**^
ERQ‐ Expressive suppression	−0.10	0.12^*^	−0.71^**^	−0.11^**^
ERQ‐ Cognitive reappraisal	0.19^**^	0.44^**^	−0.14^*^	0.24^**^

*p < 0.05.

**p < 0.01.

In addition, the relationship between the age of participants and Mentalized Affectivity was measured using Pearson's correlation coefficient, and the relationship between demographic variables of gender, field of study, educational degree, marital status, and number of children with Mentalized Affectivity was measured by Spearman's correlation coefficient. The results of these analyses are presented in Table 5. Convergent validity was demonstrated with small to moderate bivariate correlations between Identifying and the age of participants, gender, and degree; and Expressing and the age of participants, gender, marital status, and number of children (see Table 5).

**TABLE 5:** Bivariate correlations between some demographic variables and MAS subscales in the second group (*N*_2_ = 450).

	Identifying	Processing	Expressing	Recognizing
Age	0.119^*^	0.027	0.147^**^	−0.004
Gender	0.116^*^	−0.079	0.227^**^	0.050
Field	−0.053	0.011	−0.089	−0.015
Degree	0.193^**^	0.020	0.085	0.047

*p < 0.05.

**p < 0.01.

## 4 Discussion

Mentalization has long been recognized as a central determinant of psychological health. In this context, the MAS (Greenberg et al. 2017) was introduced as a self‐report measure capturing the integration of emotion regulation and mentalization. The present study extends this work by validating a Persian version of MAS in an Iranian community sample. Our analyses support a four‐factor solution—Identifying, Processing, Expressing, and Recognizing Emotions. While the first three dimensions parallel the structure proposed by Greenberg et al. (2017), the emergence of a distinct Recognizing factor suggests a culturally specific differentiation in the Iranian context.

### 4.1 Theoretical and Cultural Implications

To clarify the constructs, we conceptualize the four dimensions as follows: Identifying reflects contextualized and autobiographical appraisal of affective experience; Processing reflects regulatory modulation of intensity, duration, and expression; Expressing reflects the outward communication of affect in interpersonal contexts; and Recognizing reflects perceptual–discriminative labeling (momentary noticing and verbal tagging). The separation of Recognizing from Identifying and Processing likely reflects a culturally specific differentiation—that is, momentary labeling may constitute a separable capacity from reflective integration and regulation in this sample. We treat this as a hypothesis, not a definitive claim: targeted follow‐up (e.g., cognitive interviews, measurement‐invariance tests, and behavioral tasks) is needed to adjudicate whether this distinction generalizes beyond the present sample. Consistent with a contextual account, some items exhibited cross‐cultural shifts in loading patterns.

The concept of Identifying involves deeper complexities that include understanding emotions in the context of personal history and exploring the meaning of emotions (e.g., why do I feel that way?). The Processing Emotions factor, which includes modulating/regulating emotions, involves changing emotions regarding their duration, intensity, or frequency (Jurist 2018). Expression of Emotions leads to interpersonal interactions with others and the environment, which include getting feedback from others as well as experiencing new situations and events (Greenberg et al. 2017). Moreover, the fourth factor, Recognizing Emotions, includes recognizing and labeling emotions. In the original version of the scale, items under the Emotion Recognition and Processing Emotions factors were categorized as a single factor. Thus, these two factors are closely related so that they form a common structure. In Western culture, processing seems to be tied to awareness and recognition, and one can process and regulate his/her emotions as long as he/she can recognize them (Mauss and Gross 2004; Murata et al. 2013; Ma et al. 2018). The differentiation made between these two factors during the validation of the Persian version indicates that they do not necessarily represent a common variable and do not change randomly. It can be assumed that in the Iranian culture, there are times when people are not aware of their emotions; nevertheless, they regulate, manage, and control them. This issue can be rooted in the emotional discipline in the Iranian culture, which mainly emphasizes the management and control of emotions and is less focused on awareness and recognition of them. It might be characteristic of Asian cultures in which people tend to suppress their emotions (Butler et al. 2007; Kitayama et al. 2006). Dadkhah and Shirinbayan (2014) also showed that Iranian children tend to hide and suppress their emotions. Thus, processing emotions does not necessarily include recognizing them in Iranian culture.

This cultural distinction can also be considered through the levels of emotional awareness theory (Lane and Smith 2021). According to this theory, emotional awareness occurs at both implicit and explicit levels. There is no conscious awareness of emotions at the implicit or preconscious level, and emotional arousal is expressed in the form of bodily feelings and action tendencies. Conscious awareness of emotions exists at the explicit level, and their expression takes place through verbal and emotional processes. Studies in Western cultures show that explicit and conscious awareness is closely related to and is a precondition for adaptive emotion regulation strategies (Subic‐Wrana et al. 2014). This can be due to formal education that emphasizes explicit emotional awareness, whereas, in Iranian culture, there is an awareness at the implicit level due to the informal learning about emotions that takes place in the context.

According to Jurist and Sosa (2019), expressing emotions in East Asian cultures would not be constructive, as there is an expectation that others who are close to you will know what you are feeling; hence, needing to come out and tell them shades into having a negative valence. As a result, people need less labeling and recognition of their emotions because they do not express their emotions verbally. This can be a reason for not experiencing emotions in the second level of emotional awareness and having a physical and preconscious experience of emotions. In addition, in collectivist cultures, the process of emotion regulation focuses on protecting important people and others. Hence, processing is the priority. Therefore, we may encounter a phenomenon called emotion regulation without awareness. In individualistic cultures, this phenomenon rarely occurs due to the main attention of people on themselves (Aival‐Naveh et al. 2019). Our findings partially parallel the Italian results in showing a close relationship between processing and identifying components. However, in the Italian study, these were not merely subdivisions of a single factor: Rinaldi et al. (2021) identified them as distinct dimensions, alongside *Curiosity about Emotions* and *Autobiographical Memory*, which were absent from the original English version. The latter reflects a narrative, past‐oriented component that may vary in prominence across cultures.

Assigning some items to different factors is a notable result of the present study. Items 24 (When I express my emotions to others, it is usually jumbled) and 39 (People get confused when I try to express my emotions) were loaded by the Expression of Emotions factor. While in the original version, these items belong to the Processing Emotions factor. Considering the meaning of these items, this result can be logical because both items include the expression of people's own emotions.

### 4.2 Psychometric Evaluation and Construct Validity

To evaluate the reliability, concurrent and convergent validity of MAS, the associations between MAS, RFQ, and ERQ were examined. Regarding RFQ, the results show a positive relationship between Identifying Emotions, Processing Emotions, and Recognizing Emotions factors with certainty, and a negative relationship between Processing Emotions and Recognizing Emotions factors and uncertainty. Moreover, the results point out that the impact of Recognizing Emotions and Processing Emotions factors on certainty is more prominent. Recognizing, Processing, and Identifying were positively correlated with RFQ‐Certainty and negatively with RFQ‐Uncertainty, with the most potent effects for Recognizing and Processing. These associations suggest that accurate momentary recognition of affect and reflective elaboration of emotional experience are linked to greater epistemic access or reflective clarity about mental states, thereby supporting adaptive emotion regulation. Although Jurist's (2018) theoretical account emphasizes reflective clarity, autobiographical re‐evaluation, and epistemic access, it should not be treated as identical to the RFQ's operationalization of “certainty.” Instead, the RFQ correlations provide convergent but not interchangeable evidence for these constructs.

A positive correlation was observed between cognitive reappraisal and three of the four factors of MAS (i.e., Identifying Emotions, Processing Emotions, and Recognizing Emotions). In contrast, there is a negative correlation between Cognitive reappraisal and Expression of emotions. We can assume that all factors related to cognitive reappraisal refer to an individual's inner abilities (i.e., Identifying, Processing, and Recognizing Emotions) (Greenberg et al. 2017), and the Processing Emotions factor has the strongest positive relationship with cognitive reappraisal, because cognitive reappraisal is a part of emotional regulation mechanisms (Jurist 2018; Gross 2024).

In the present study, gender showed a small‐to‐moderate association with Expressing (*r* = 0.227, *p* < 0.01), suggesting that female participants reported slightly higher expressive tendencies. This pattern aligns with general evidence from cross‐cultural research indicating that women often report greater emotional expressivity than men, likely reflecting socialization and normative expectations. Given the female majority in our sample and the online recruitment method, this finding should be interpreted cautiously and warrants replication in more balanced samples.

### 4.3 Limitations and Future Directions

This study has several limitations that constrain its generalizability and point to clear priorities for future research. First, the sample consisted of nonclinical community participants recruited online through snowball sampling, with women overrepresented. Such a convenience sample may distort correlation patterns and restrict the strength of population‐level inferences. Future research should therefore employ probability‐based or quota‐stratified sampling, ensure balanced gender and age distributions, and replicate findings in independent regional cohorts—including clinical populations—while also testing measurement invariance across gender, age, and clinical status. The instrument retained 52 items. Although internal consistency was high, AVE values below 0.50 indicate that several indicators capture only modest levels of unique variance. A focused program of content refinement and short‐form development is warranted: low‐performing items should be rewritten using cognitive interviewing, evaluated with modern psychometric approaches (e.g., graded‐response IRT), and validated in shorter forms through CFA/SEM in independent samples. In addition, future work should establish test–retest reliability, assess longitudinal and therapeutic sensitivity to change, and examine potential response‐style artifacts (e.g., effects of RK content).

External validity should be extended beyond basic convergent associations. We recommend evaluating incremental validity relative to adjacent constructs (e.g., alexithymia, broad emotion‐regulation composites, personality), incorporating multi‐method criteria (behavioral or performance‐based affect‐labeling tasks, clinician ratings, and physiological indices), and testing predictive and clinical utility (e.g., response to mentalization‐based or emotion‐focused interventions). Although this validation was conducted with a nonclinical sample, the Persian MAS shows considerable promise for clinical use—a claim that remains provisional. Future studies should examine diagnostic sensitivity and specificity, establish preliminary norms and thresholds for clinically meaningful change, and test whether MAS scores can prospectively predict treatment engagement or outcomes.

## 5 Conclusion

This study provides empirical support for the psychometric validity and cultural adaptation of the Persian version of the MAS in an Iranian sample. Findings revealed a four‐factor structure—Identifying Emotions, Processing Emotions, Expressing Emotions, and Recognizing Emotions—where the latter emerged as a culturally distinct factor. This distinction underscores potential cultural variations in emotional awareness and regulation, particularly the tendency in Iranian culture to regulate emotions without explicit recognition. The scale demonstrated strong internal consistency, concurrent validity, and convergent validity with related constructs, reinforcing its utility as a reliable tool for assessing mentalized affectivity. Despite limitations such as the absence of a clinical sample and gender imbalance, the results support the MAS as a promising instrument for research and clinical applications in Persian‐speaking populations. Future studies should further explore its applicability in clinical contexts and diverse demographic groups to enhance its generalizability. By contributing to the cross‐cultural validation of the MAS, this study facilitates further research on mentalization in non‐Western populations, broadening the scope of affectivity assessment across cultural contexts.

## Author Contributions

**Fateme Jafarpoor**: conceptualization, formal analysis, methodology, visualization, project administration, validation, writing – review & editing, writing – original draft. **Elmira Shayegh**: data curation, investigation, writing – review & editing. **Parvin Haghjoo**: data curation, investigation, writing – review & editing. **Saeed Ghanbari**: conceptualization, methodology, supervision, writing – review & editing.

## Peer Review

The peer review history for this article is available at https://publons.com/publon/10.1002/brb3.70880.

## Supporting information




**Supplementary Figure**: brb370880‐sup‐0001‐FigureS1.docx

## Data Availability

The data that support the findings of this study are available from the corresponding author upon reasonable request.
