# Supplementary figures and images for: Persian Mentalized Affectivity Scale (MAS): Reliability, Validity, and Cultural Considerations
Source: Brain Behav. 2025 Sep 21;15(9):e70880. doi: 10.1002/brb3.70880 (PMC12451027; doi:10.1002/brb3.70880)

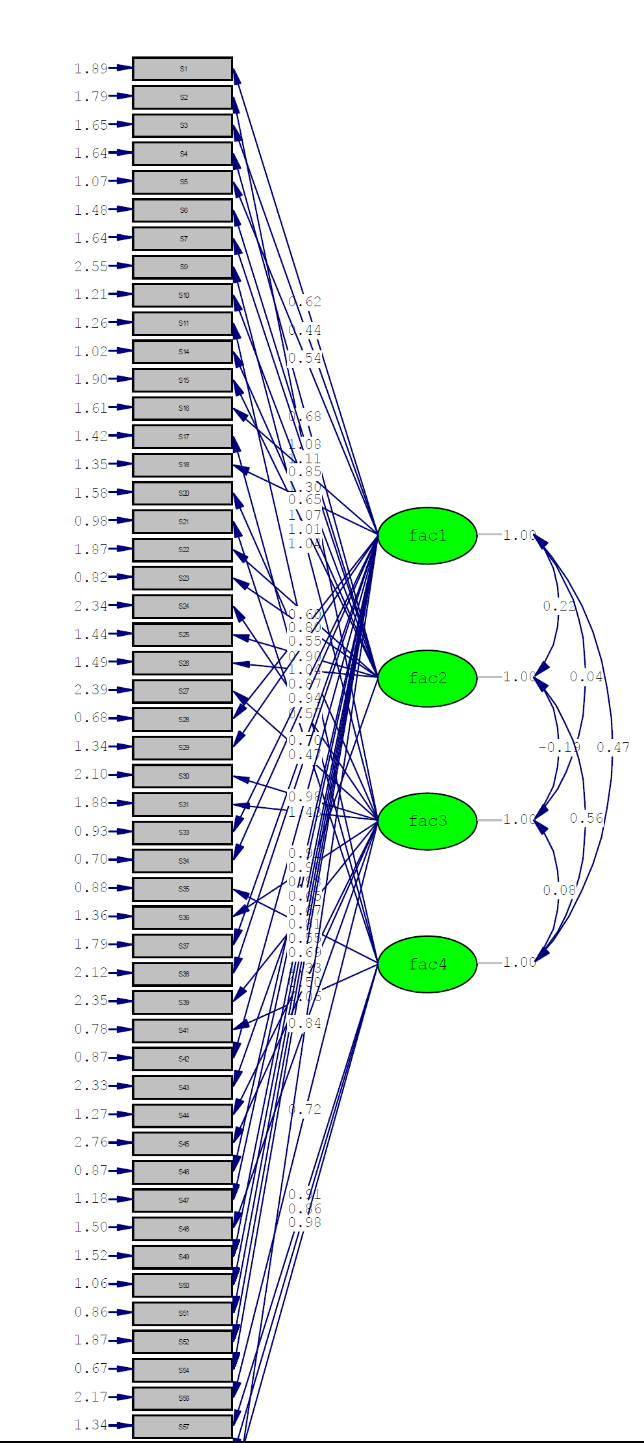


**Fig. S1. The 4-factor structure of 52-item MAS**

Supplement: Supplementary file 1 — Supplementary Figure: brb370880‐sup‐0001‐FigureS1.docx [file BRB3-15-e70880-s001.docx]
